# Network pharmacology and transcriptomic profiling elucidate the therapeutic effects of *Ranunculus ternatus* Thunb on liver fibrosis via MK3-NF-κB inhibition

**DOI:** 10.18632/aging.205629

**Published:** 2024-03-08

**Authors:** Lu Han, Guoyuan Lin, Jianchao Li, Qingxiu Zhang, Tao Ran, Tao Huang, Ruihan Hu, Shu Feng, Gaoliang Zou, Shaojie Chen, Xueke Zhao

**Affiliations:** 1 Department of Infectious Diseases, The Affiliated Hospital of Guizhou Medical University, Guiyang, Guizhou Province, China; 2 Department of Gastroenterology, Guizhou Provincial People’s Hospital, Guiyang, Guizhou Province, China; 3 Department of Cardiology, Guiqian International General Hospital, Guiyang, Guizhou Province, China; 4 Department of Hepatobiliary Surgery, The Affiliated Hospital of Guizhou Medical University, Guiyang, Guizhou, China

**Keywords:** Liver fibrosis, β-sitosterol, network pharmacology, transcriptomics, MAPK Activated Protein Kinase 3

## Abstract

Activation of hepatic stellate cells (HSCs) is critical in the progression of liver fibrosis and is a promising target for anti-hepatic fibrosis drug development. Moreover, effective pharmacological interventions targeting this pathomechanism are scarce. Our study confirms the therapeutic value of β-sitosterol, a major constituent of *Ranunculus ternatus* Thunb, in hepatic fibrosis and identifies its underlying mechanisms. After treatment with β-sitosterol, CCL4-induced hepatic fibrosis was reversed in mice, while inflammatory and hepatic fibrosis indices were improved. Meanwhile, we explored the molecular mechanism of β-sitosterol treatment for hepatic fibrosis and, based on RNA-seq results, found that the ameliorative effect of β-sitosterol on hepatic fibrosis was associated with the MK3 and NF-κB signalling pathways. MK3, an important kinase in the MAPK pathway, plays a role in transmitting upstream and downstream signals, whereas the NF-κB signalling pathway has been shown to be associated with HSC activation. We verified the interaction between MK3 and IκB in HSC cells using endogenous Co-IP, whereas β-sitosterol reduced the binding of MK3 to IκB and the activation of the NF-κB signalling pathway. Our findings reveal the mechanism of β-sitosterol in the treatment of liver fibrosis, suggesting that β-sitosterol may be a promising drug for the treatment of liver fibrosis and deserves further investigation.

## INTRODUCTION

Liver fibrosis is a long-term liver disorder which involves the excessive growth and buildup of fibrous tissue. This leads to structural damage and impaired liver function [1]. The activation of hepatic stellate cells (HSCs) is a key event in the evolution of this condition [2]. When exposed to inflammation, non-alcoholic fatty liver disease, alcohol consumption, viral infections, drug-induced toxicity, and other agents, quiet HSCs (qHSCs) are activated and transformed into activated HSCs (aHSCs). aHSCs produce an increased amount of components of the extracellular matrix (ECM) [3]. Deposition of ECM unchecked can lead to liver fibrosis, cirrhosis, and liver failure [4, 5]. Promoting apoptosis of HSCs represents an ideal strategy for clearing activated cells without causing inflammatory damage to the liver, making it a primary goal for effective anti-fibrotic therapies [6].

Treatment of liver fibrosis and prevention of its progression are limited to a few drugs [7, 8], highlighting the imperative need for novel interventions to combat this pathology and offer hope for improved clinical outcomes.

*Ranunculus ternatus* Thunb (RTT) is a climbing plant belonging to *Ranunculaceae* family. Its root, RTT, possesses a remarkable medicinal potential and is widely used in traditional Chinese medicine. This botanical marvel contains various active constituents, including flavonoids, endolipids, and sterols, each with distinct pharmacological properties such as antioxidant, anti-inflammatory, hypoglycemic, and hypolipidemic activities [9]. RTT has gained attention for its efficacy in treating various diseases, including hepatitis, renal fibrosis, and some types of cancer [9]. In Chinese medicine, this herb has been utilized as a potent remedy against hepatitis B [10]. Additionally, RTT has exhibited noteworthy immunomodulatory and hepatoprotective effects in mouse models [11]. An effective treatment for various liver diseases has been found in *Ranunculus ternatus* Thunb, all of which are causative agents of liver fibrosis, so we wanted to investigate whether *Ranunculus ternatus* Thunb could alleviate the progression of liver fibrosis. However, the underlying mechanisms responsible for these therapeutic effects require further elucidation, warranting additional research.

Network pharmacology is a comprehensive approach in drug research that integrates network analysis and systems biology [12]. It combines information from different levels, including drug molecules, proteins, and genomes, to construct intricate network models. This method enables the exploration of crucial aspects such as mechanisms of drug action, potential side effects, and drug targets. Network pharmacology is widely employed in novel drug discovery, drug repurposing, and drug safety assessment, offering great promise for therapeutic interventions [13]. This approach not only expedites drug development but also provides more precise and effective treatment alternatives for various diseases.

RTT’s potential for treating liver fibrosis was examined using a network pharmacology approach. With Traditional Chinese Medicine Systems Pharmacology Database and Analysis Platform (TCMSP), we analyzed RTT’s key bioactive components and predicted their molecular targets. Additionally, we investigated disease-related databases such as GeneCards, OMIM, and TTD to identify gene targets associated with liver fibrosis. To explore the mechanisms behind the therapeutic effects of RTT in hepatic fibrosis, we applied enrichment analysis with Gene Ontology (GO) and Kyoto Encyclopedia of Genes and Genomes (KEGG). Employing a protein-protein interaction (PPI) network strategy, we identified hub networks and gene targets of RTT that can be used to treat liver fibrosis. Our computational findings were validated *in vitro* and *in vivo*. RNA-seq analysis of mouse liver samples was done to gain insight into the intricate processes underlying the therapeutic effects of β-sitosterol, the active element of RTT herb. Bioinformatics analysis uncovered that β-sitosterol can obstruct the triggering of the NF-κB pathway in LX-2 cells, resulting in the death of HSCs, by targeting MK3. This could effectively reduce the onset and progression of liver fibrosis.

## MATERIALS AND METHODS

### Identification of the principal constituents within RTT

**Figure 1:**
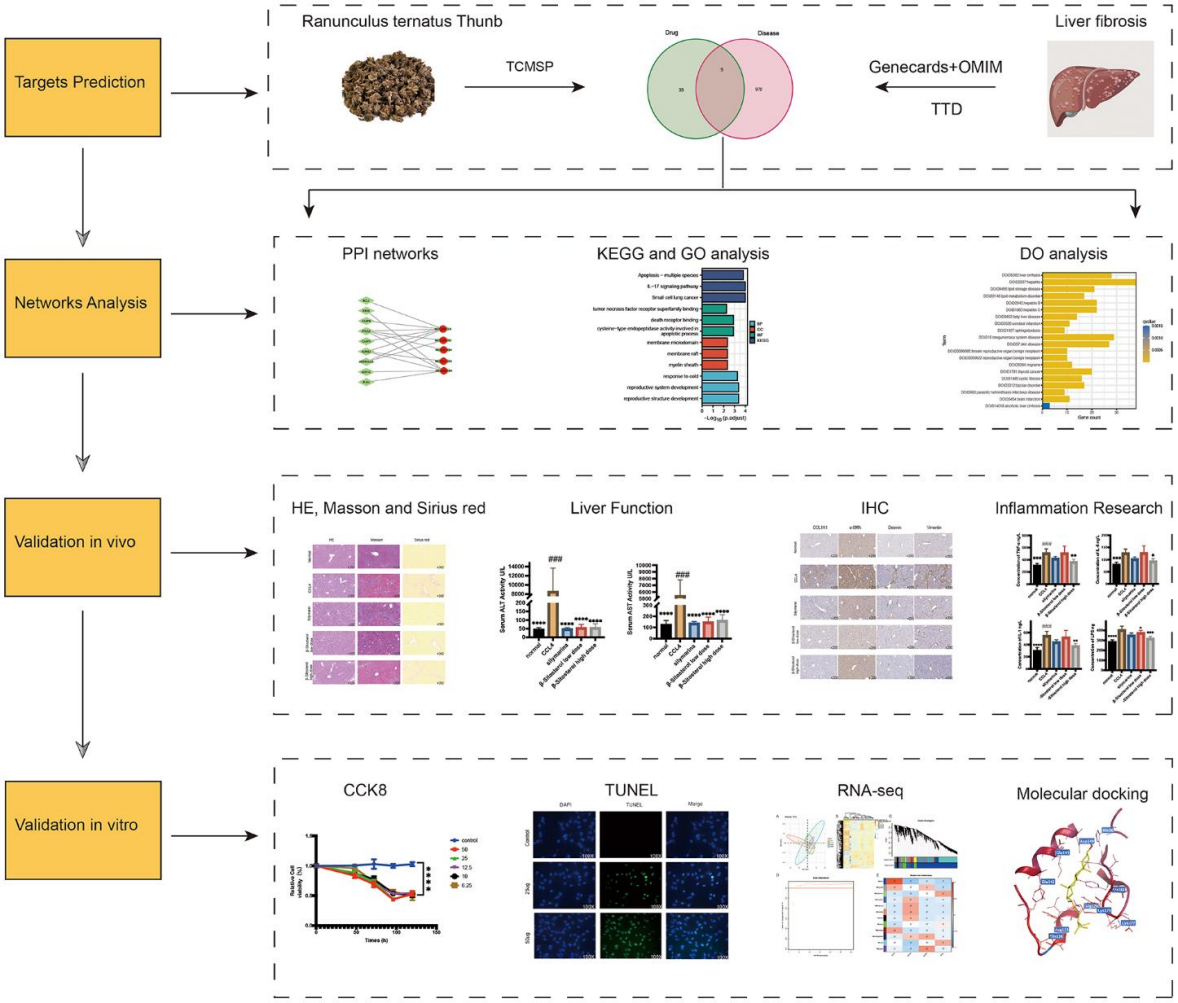
Flow chart of the experiment.

In this study, we determined the key constituents of RTT utilizing the TCMSP (https://old.tcmsp-e.com/tcmsp.php) [14]. We focused on compounds with an oral bioavailability of at least 30%, considering their pharmacokinetic properties of absorption, distribution, metabolism, and excretion [15]. To ensure pharmacological relevance, compounds demonstrating a drug-likeness index equal to or surpassing 0.18 [16] were considered therapeutically active entities. Figure 1 depicts the flow chart for the study.

### Predicting genes targeted by RTT for the amelioration of liver fibrosis

To identify potential gene targets influenced by RTT for the therapeutic management of liver fibrosis, we employed the keyword “liver fibrosis” to gather disease-related targets from reputable databases including the GeneCards database (https://www.genecards.org/) [17], the Online Mendelian Inheritance in Man (OMIM) database (https://www.omim.org/) [18], and the Therapeutic Target Database (TTD) (http://db.idrblab.net/ttd) [19]. We compared the targets associated with the active constituents of RTT with those associated with liver fibrosis. This approach enabled us to identify potential gene targets of RTT, unveiling its efficacy in combating liver fibrosis.

### Constructing a network of active compound-disease-target interactions

Employing Cytoscape 3.8.0 software [20], we integrated active compounds and their respective targets to form a comprehensive compound-disease-target interaction network, and graphically depicted this network in an effort to comprehend its intricate structure. Furthermore, we employed the Network Analyzer tool to extract valuable insights from the network analysis and gain a deeper understanding of its structural and functional characteristics.

### Enrichment assessment using GO and KEGG

To elucidate the functional implications of our findings, we employed the ClusterProfiler package in R [21, 22]. This tool allowed us to annotate and visualize KEGG pathways and GO terms encompassing Biological Processes (BP), Cellular Components (CC) and Molecular Functions (MF). By utilizing this strong analytical framework, we aimed to unravel the complex associations and biological significance of the observed molecular interactions and pathways.

### Identification of potential targets

To investigate the complex connections between potential targets, the Search Tool for Retrieval of Interacting Genes or Proteins (STRING) database was used for the analysis (https://www.string-db.org) [23]. This comprehensive resource provided valuable insights into the PPIs underlying these targets. The species was set to *Homo sapiens*, ensuring relevance to human biology. Using a minimum interaction score of 0.4, we filtered PPI network maps for simplicity. The resulting TSV files were seamlessly imported into Cytoscape 3.8.0 from the STRING database.

### Docking analysis of molecules

The Protein Data Bank (PDB ID 481107734) in PDB format provided us with the three-dimensional structure of the target, and PubChem yielded a structure for sitosterol. The rotatable bonds of the compound were determined using Autodock Tools 1.5.6. To ensure the accuracy of the docking, water molecules, hydrogen atoms, and charges were carefully removed from the protein structure. Subsequently, we conducted molecular docking using the MOE software. The binding energy analysis allowed us to assess the strength of the intermolecular interactions between the compound and its target, providing valuable insights into their binding activity.

### Reagents and antibodies

The CCl4 and corn oil were purchased from Sigma-Aldrich (St. Louis, MO, USA). β-sitosterol was procured from MedChemExpress (HY-N0171A) (USA). Anti-collagen type I alpha 1 chain (COLA1A EPR22894-89) antibody was purchased from Abcam (Waltham, MA, USA), and from Proteintech, China, we purchased p50 (15506-1-AP) and smooth muscle actin (14395-1-AP) antibodies. The antibodies against MAPK-activated protein kinase 3 (MAPKAPK3) (R27029), p65 (R25149), p-p65 (R380738), cleaved caspase (c-CASP) 3 (R380169), c-CASP8 (250106), CASP3 (R23315), and CASP8 (R381304), Desmin (R380843) and Vimentin (R50015) were purchased from ZenBio (China). Mmbio, China supplied the serotonin enzyme-linked immunosorbent assay (ELISA) kit. The TUNEL kit was obtained from Elabscience, China (E-CK-A420). The Total Protein Extraction kit was purchased from Abbkine, China (KTP3006).

### The culture of cells

Obtained from Wuhan Pricella in China, LX-2 cell lines were carefully grown in the most suitable environment. The cells were grown in a Dulbecco’s Modified Eagle medium with high glucose content, together with 10% fetal bovine serum (Wuhan Pricella, China), as well as penicillin (100 U/mL; Gibco, Waltham, MA, USA) and streptomycin (100 μg/mL; Gibco) antibiotics. To ensure optimal growth and viability, the cells were kept in an environment with 5% CO_2_ at a temperature of 37° C.

### Cell viability and proliferation

The viability of LX-2 cells following β-sitosterol exposure was evaluated using the CCK-8 assay (ApexBio, Houston, TX, USA). In commencing the experiment, 4 x 10^3 cells (100 μL/well) were placed in 96-well plates with the aim of maintaining a consistent cell density for all samples. Afterward, the plates were put in a regulated incubator with a temperature of 37° C and a CO_2_ level of 5%, providing the ideal pre-incubation period of 24 hours.

Evaluating the effect of β-sitosterol, five concentrations (50 μg/mL, 25 μg/mL, 12.5 μg/mL, 10 μg/mL, and 6.25 μg/mL) were put into the respective wells. The control group was exposed to the corresponding solvent. Following incubation periods of 48, 72, 96, and 120 hours, 10 μL of CCK-8 solution was added to each well and the plates were left to incubate for an extra 4 hours. The plates were subsequently analyzed spectrophotometrically, with the absorbance being measured at 450 nm.

### Animals

Animal experimentation was conducted in compliance with the ARRIVE guidelines and was granted ethical approval from the Guizhou Medical University Ethics Committee (No. 2200650). C57BL/6 male mice of age 7-8 weeks were obtained from Chongqing Tengxin Laboratory Animal Co., located in Chongqing, China, for the scientific investigation. The well-being of the mice was of utmost importance, and they were housed in controlled laboratory rooms under optimal conditions, ensuring a temperature range of 23 - 25° C, a balanced 12-hour light/dark cycle, and a humidity level of 55% ± 5%. Throughout their stay, water and an approved standard chow were available ad libitum to the mice. A minimum acclimatization period of seven days was provided prior to any experimental procedures to ensure their physiological equilibrium.

### Experimental protocol

Randomly, thirty mice were divided into five groups of six each: control, CCl4, silymarin (SIL), and β-sitosterol in low and high doses. The control group was injected intraperitoneally with peanut oil, and all other groups were injected intraperitoneally with 10% CCl4 (15 μL/g) three times a week for a fortnight. Starting from the third week, the CCl4 group received saline by gavage, while the β-sitosterol low dose, β-sitosterol high dose, and SIL groups received daily oral doses of β-sitosterol at 3.5 mg/kg, β-sitosterol at 7 mg/kg, and a four-week course of silymarin (SIL) at 160 mg/kg body weight. The doses of β-sitosterol and SIL were determined based on previous studies [24]. Both β-sitosterol and SIL were dissolved in saline for oral administration. The body weights of mice were recorded weekly throughout the entire duration of the study. Total time is 6 weeks, in the seventh week, isoflurane 2% was used to anesthetize the mice deeply, and plasma samples were collected via orbital blood sampling.

### Protein extraction and Western blot

LX-2 cells underwent a 48-hour exposure to β-sitosterol concentrations of 0, 6.25, 10, 12.5, 25, and 50 μg/mL. Then, the cells were given two rinses with a PBS solution containing 0.01% calcium and magnesium ions. The proteins were extracted utilizing the Abbkine Total Protein Extraction Kit, in accordance with the instructions of the manufacturer. The bicinchoninic acid assay was utilized to quantify the concentration of the proteins that were extracted. As previously described, LX-2 cells were lysed and proteins were analyzed by Western blotting [25].

### Histopathological studies

Mice livers were embedded in paraffin after being treated with 4% formaldehyde, washed, dehydrated, and formaldehyde treated. Paraffin sections of 4-5 μm thickness were subjected to staining with hematoxylin and eosin (from Beijing Huayueyang Biotechnology Co., Ltd., China), Sirius Red (from Beijing Lejin Biotechnology Co., Ltd., China), and Masson (from Solebro Biotechnology Priority, Inc., China). Subsequently, morphological features and fibrosis were observed using orthomosaic microscopes (Zeiss microscope Axio imager, Germany) at magnifications of 20X and 40X.

### Immunohistochemistry (IHC) staining

Paraformaldehyde (4%) was used to fix the liver samples, and then they were embedded in paraffin for IHC. Sections were subjected to antibody staining, using horseradish peroxidase-conjugated secondary antibodies. Following staining with a diaminobenzidine colorimetric reagent and counterstaining with hematoxylin, representative images were obtained by scanning the slides (Zeiss microscope Axio imager, Germany). The area ratio of protein expression in the livers of each group of mice was determined using ImageJ software.

### TUNEL staining

Following a 24-hour incubation of 4x10^4 cells on 8-well chamber slides, fixation was carried out using a 4% paraformaldehyde solution at 24° C for 20 minutes. Subsequently, a 5-minute gentle wash with PBS was performed. To augment cellular permeabilization, 0.5% Triton X-100 was incorporated and incubated for 30 minutes.

For one hour, the samples were maintained at 37° C and incubated with a 50 μL TUNEL reaction mixture. Following two 5-minute washes in PBS, the nuclei were stained with 4’,6-diamidino-2-phenylindole (DAPI, Molecular Probes/Invitrogen, Waltham, MA, USA) diluted in PBS for a 15-minute period at 24° C. Two 5-minute washes with PBS were performed, and coverslips were carefully positioned on the slides. Imaging was conducted utilizing an ortho-fluorescent microscope from Zeiss microscope Axio Imager A2 (Zeiss microscope Axio imager, Germany).

### Inflammation assessment

After centrifuging the plasma at 3000 rpm for 5 minutes, the supernatant was collected to analyze the inflammatory status. Plasma lipopolysaccharide (LPS), tumor necrosis factor-alpha (TNF-α), interleukins 1 (IL-1) and 6 (IL-6) levels of essential inflammatory markers were evaluated using ELISA kits, following the manufacturer’s instructions.

### Liver function

Automation was utilized to analyze plasma samples and measure the levels of aspartate aminotransferase (AST) and alanine aminotransferase (ALT). This analysis was performed using a CD-1600CS Biochemistry Analyzer (Abbott Laboratories, Abbott Park, IL, USA).

### RNA-sequencing, differential gene analysis and WGCNA analysis

Tissue samples from mouse liver were obtained and RNA was obtained by means of TRIzol reagent (Invitrogen, Waltham, MA, USA). Subsequently, the extracted RNA underwent a rigorous assessment of integrity, purity, and quality using agarose gel electrophoresis (China), a NanoPhotometer spectrophotometer (Thermo Fisher Scientific, Waltham, MA, USA), and an Agilent 2100 bioanalyzer (Agilent Technologies Co., Ltd., Santa Clara, CA, USA).

The NEBNext® UltraTM RNA Library Prep Kit was employed for library construction, involving the enrichment of polyA mRNA and cDNA synthesis. After this, a NovaSeq 6000 sequencer (Illumina, San Diego, CA, USA) was used to execute paired-end sequencing of 150 bp. Quality control was considered during data analysis. Adapter sequences were removed, reads with N bases or low quality were discarded, and Q20, Q30, and GC content metrics were computed for clean data. These steps ensured the generation of high-quality data for subsequent analysis.

Differential expression analyses between the two comparison combinations (4 biological replicates per group) were performed using DESeq2 software (1.16.1). DESeq2 provides statistical procedures for determining differential expression in numerical gene expression data using models based on negative binomial distributions. Genes with P values <0.05 calculated by DESeq2 were defined as differentially expressed. The corrected P-value was used as thresholds for significant differential expression.

For gene co-expression analysis, this experiment utilizes the common systems biology algorithm WGCNA. After clustering the genes in the samples using the hclust function against the WGCNA package in the R software, outliers were detected and removed. Following the removal of outliers, clustering was conducted based on the gene expression level of each sample to uncover the correlation between samples. After analyzing the correlation of expression levels between genes, a gene clustering tree was constructed, and co-expression modules were identified through dynamic pruning. A module was required to contain a minimum of 50 genes. Subsequently, modules showing comparable expression patterns were consolidated by assessing the similarity of their module eigenvalues, using a threshold of 0.75. Each sample’s expression pattern of the module genes was indicated by the module eigenvalue MEmagenta, and the heat map of the sample expression pattern was created using the sample eigenvalues.

### Co-immunoprecipitation (Co-IP) assay

LX-2 cells treated with β-sitosterol or solvent treated were harvested and the protein was extracted using the Abbkine kit as described previously, the supernatant was collected and the concentrated protein was quantified. 1 mg of protein was taken with MK3 (Abcam, EPR13969) and IκB (Proteintech, China, 10268-1-AP) The monoclonal antibodies were incubated overnight at 4° C in a spin. At the same time, a monoclonal antibody against IgG (Beyotime, China, A7016) was used as a negative control. Fifty microliters of immunoprecipitation beads (Yeasen, China, 36417ES08) were placed into each lysate, left to incubate for 4 hours, and then washed three times with lysis buffer. Afterward, the pellets were suspended in SDS-PAGE loading buffer, boiled for 8 min, and then separated on SDS-PAGE for protein analysis using either anti-IκB or anti-MK3 antibodies.

### Statistical analysis

GraphPad Prism 9.0 software was utilized to conduct statistical analysis. To compare two groups, a two-tailed Student’s t-test was utilized when the data had a normal distribution. To compare multiple groups of data with a normal distribution, one-way ANOVA was utilized. The results, which were expressed as mean ± standard deviation, were considered statistically significant if the P level was below 0.05.

### Data availability statement

The datasets used and analysed during the current study are available from the corresponding author on reasonable request.

## RESULTS

### Disease targets for liver fibrosis and active ingredients of RTT

The TCMSP database was explored to recognize the active ingredients and therapeutic targets of RTT. To select the active ingredients, criteria of oral bioavailability of at least 30% along with drug-likeness of 0.18 or more were applied, leading to a list of 10 active ingredients, mainly composed of phytosterols (Supplementary Table 1).

**Figure 2:**
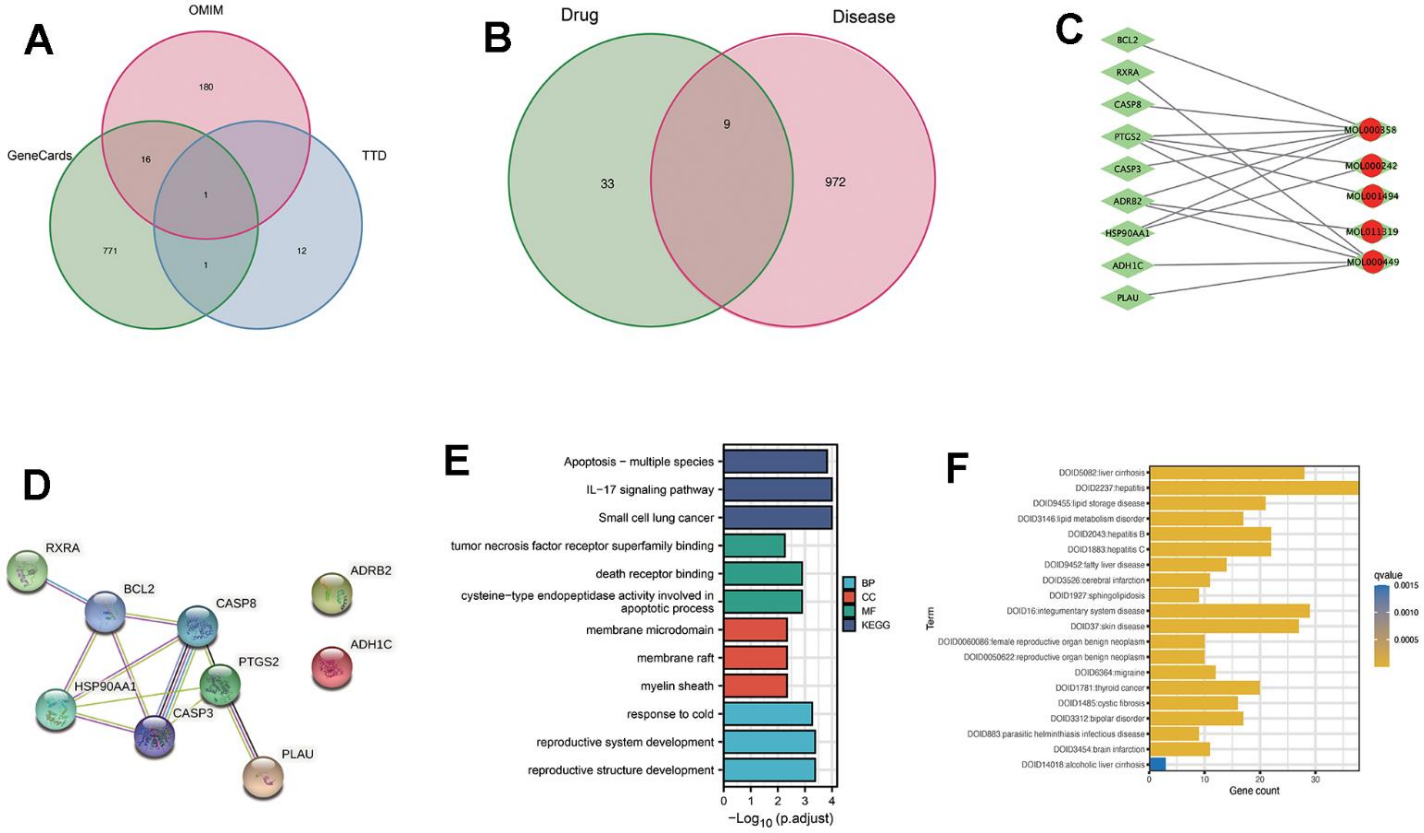
**Analysis of the active ingredients of *Ranunculus ternatus* Thunb and liver fibrosis causative genes through network pharmacology.** (**A**) Genecards, TTD and OMIM databases of disease-related genes, taken as a concatenation. (**B**) Drug targets and disease targets taken as intersection results. (**C**) Compound-target network. Red dots represent activities contained in cataplasma. Green rhombus represents targets. (**D**) Set the minimum required interaction score to 0.4 and draw a PPI network diagram. (**E**) Target genes were subjected to GO and KEGG analysis, focusing on the apoptotic pathway. (**F**) DO enrichment for the active ingredient of *Ranunculus ternatus* Thunb herb, β-sitosterol.

To expand the search for therapeutic targets of RTT in liver fibrosis treatment, relevant targets were collected from multiple databases. The targets included were 789 from GeneCards related to liver fibrosis, 197 from OMIM, and 14 from the TTD database. Following the removal of common genes, 972 genes related to liver fibrosis were retained for further analysis (Figure 2A).

By comparing the targets of the 10 active ingredients with the 972 disease-related genes, nine overlapping genes were identified (Figure 2B). These overlapping genes served as the core targets for further investigations.

### An analysis of the PPI network, GO, KEGG, and disease ontology (DO) enrichment

Following the screening, the active ingredients of RTT were determined to predominantly consist of phytosterols.

Utilizing Cytoscape 3.9.1, a network analysis was conducted to gain a better understanding of the connections between active compounds and targets, as demonstrated in Figure 2C, 2D. The network comprised nine nodes and eleven edges, with targets such as CASP3, CASP8, and BCL2 exhibiting a degree of four, indicating their significance within the network.

To gain more insight into the functional implications of the identified targets, GO and KEGG enrichment analyses were conducted, as depicted in Figure 2E.

Results of the analysis showed a major increase in the number of genes related to apoptotic and death receptor signaling pathways. Based on these findings, β-sitosterol, one of the key active ingredients, was selected for subsequent experimental validation.

To comprehend the therapeutic potential of β-sitosterol more thoroughly, a DO analysis was conducted (Figure 2F). The drug targets associated with β-sitosterol were found to be mostly associated with liver conditions such as cirrhosis, hepatitis, and non-alcoholic fatty liver disease. Consequently, β-sitosterol was further tested *in vivo*.

### β-sitosterol alleviates CCl_4_-induced liver injury in mice

**Figure 3:**
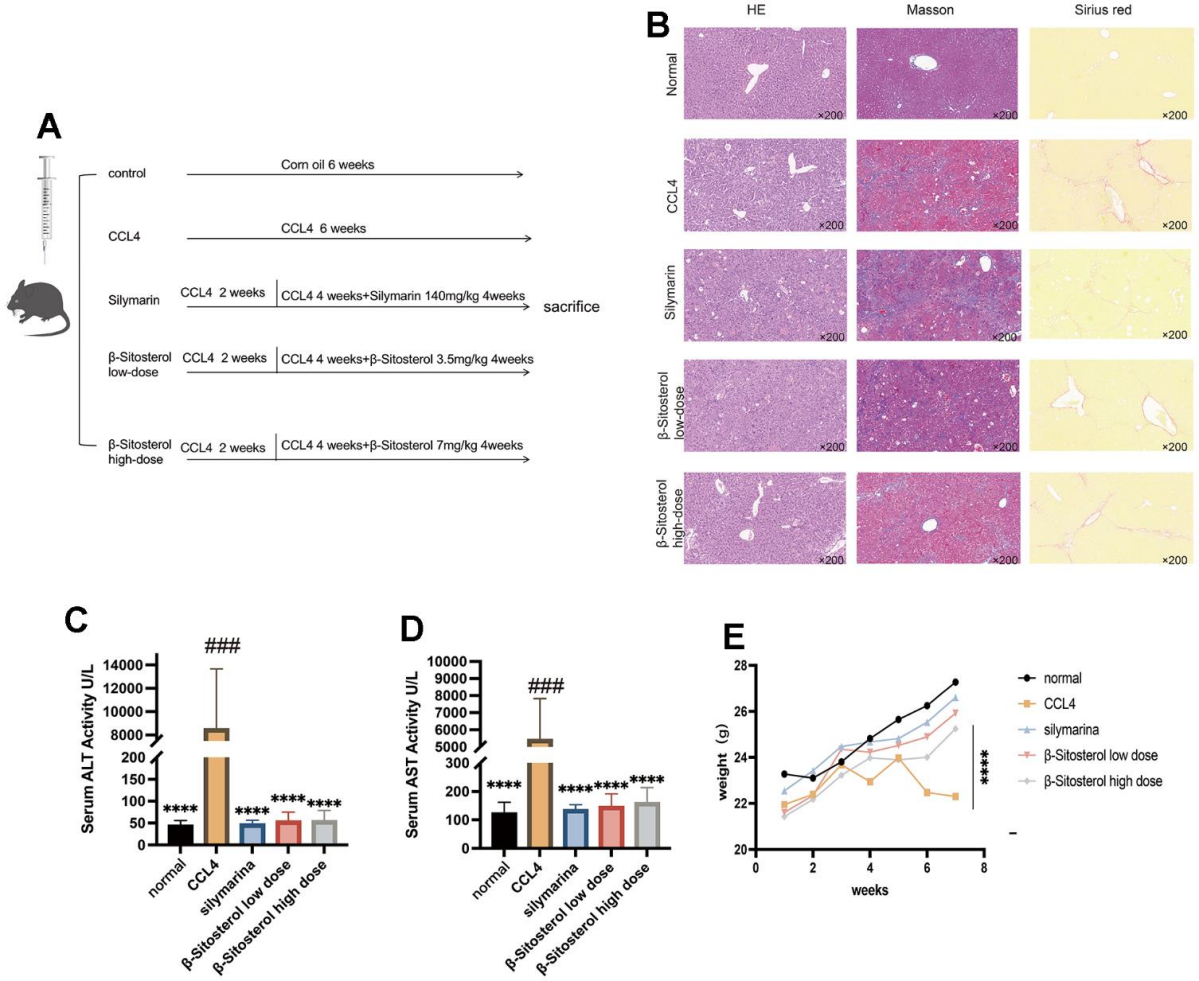
**Animal experiment flow chart and evaluation of β-sitosterol for the alleviation of CCL4 liver fibrosis in mice.** (**A**) Experimental schedule. (**B**) Liver sections stained with hematoxylin and eosin (H&E), sirius red, trichrome masson and collagen Iα are shown and observed under a light microscope. (**C**, **D**) β-sitosterol alleviates the CCl4 induced elevation in serum AST and ALT in mice. (**E**) β-sitosterol alleviates weight loss in CCL4 mice. Representative results of at least six independent experiments (biological replicates) are shown in all panels. * P <0.05, ** P <0.01, *** P <0.001, **** P <0.0001.

An experiment with animals was conducted to assess the efficacy of β-sitosterol in treating liver fibrosis (Figure 3A). Histological examination showed a well-defined portal area with no indications of swelling or infiltration of inflammatory cells in the control group.

Conversely, the CCl_4_ model group exhibited enlarged portal areas with disrupted architecture, hepatocyte ballooning, and significant infiltration of inflammatory cells. Masson’s trichrome staining revealed a notable decrease in collagen deposition in the confluent area for the control, SIL, and β-sitosterol low and high-dose groups, when compared to the CCl4 model group.

The findings from Sirius Red staining were consistent with those from Masson’s trichrome staining. β-sitosterol was found to be effective in reducing CCl4-induced liver fibrosis in mice, as seen in Figure 3B.

Analysis of the serum levels of AST and ALT in mice indicated that β-sitosterol could ameliorate CCl_4_-induced liver dysfunction in mice. Additionally, β-sitosterol demonstrated similar efficacy as silymarin in restoring the body weight of mice to normal levels, attenuating the CCl_4_-induced weight loss (Figure 3C–3E). Our results indicate that β-sitosterol can alleviate CCl_4_-induced liver damage in mice, suggesting its usefulness in treating liver fibrosis.

### β-sitosterol was found to reduce signs of liver fibrosis and inflammation in mice that had been exposed to CCl4-induced liver damage

**Figure 4:**
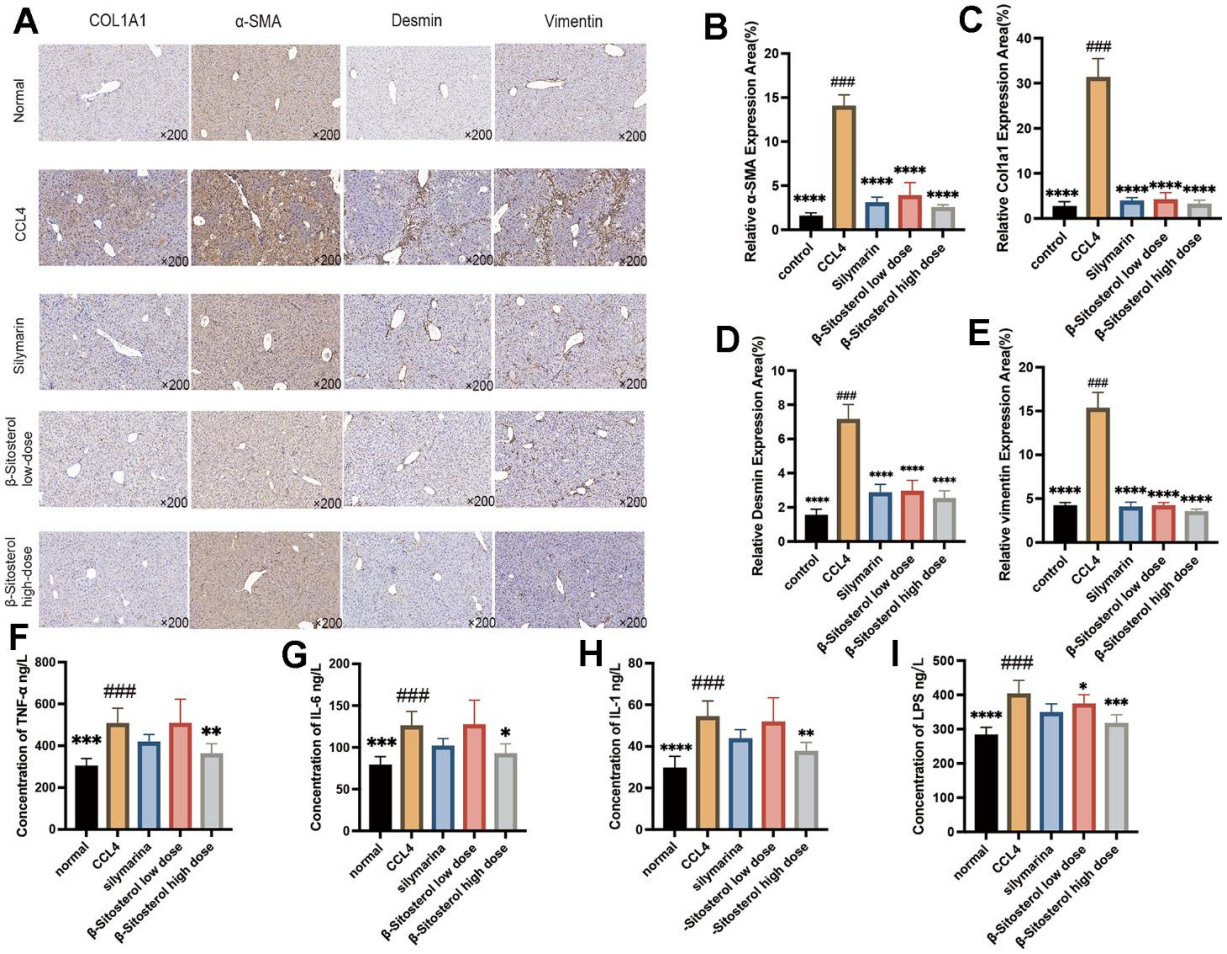
**β-sitosterol attenuates fibrosis markers and serum inflammatory markers in CCL4 model mice.** (**A**) Differential protein expression of the liver fibrosis markers COL1A1, α-SMA, Desmin and Vimentin by immunohistochemistry. (**B**–**E**) Relative expression area of fibrosis markers, showing representative results from 6 independent replicate trials (n = 6). Compared to CCL4; * P < 0.05, ** P < 0.01, *** P < 0.001, **** P < 0.0001. (**F**–**I**) Expression level of LPS, TNF-α, IL-1and IL-6 proteins in the serum of CCL4-induced mice (n=6) compared with control mice, β-sitosterol low-dose and β-sitosterol high-dose (Corn, n=6, β-sitosterol low-dose, n=6, β-sitosterol high-dose, n=6) measured by ELISA assay. Compared to CCL4; * P < 0.05, ** P < 0.01, *** P < 0.001, **** P < 0.0001.

**Figure 5:**
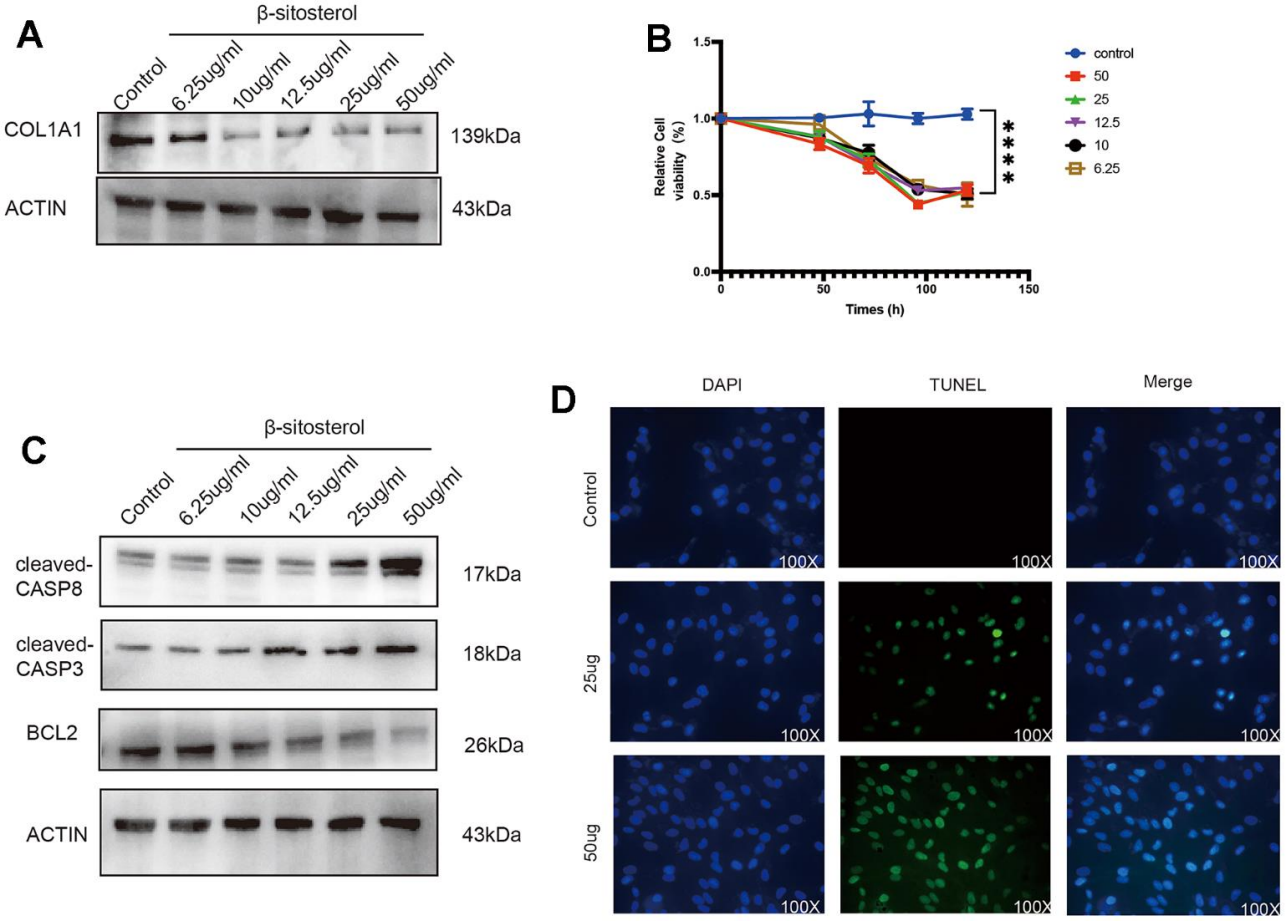
**β-sitosterol alleviates liver fibrosis by promoting LX-2 apoptosis.** (**A**) COL1A1 was reduced after treating LX-2 with different doses of β-sitosterol for 48 h, showing concentration dependence. (**B**) Cellular activity was measured at different time points after treatment of LX-2 cells with different doses of β-sitosterol. (**C**) Cells treated with different doses of β-sitosterol were assayed for CASP8, cleaved-CASP8, CASP3, cleaved-CASP3 as well as BCL2 by WB. (**D**) After treatment of LX-2 cells with β-sitosterol, staining for TUNEL (green) and 4′,6-diamidino-2-phenylindole (DAPI) scale bar, 100 μm.

To assess the expression of four markers of liver fibrosis (COL1A1, α-SMA, Desmin, and Vimentin) and to investigate the possibility of β-sitosterol in reducing liver fibrosis, IHC staining was performed. Fibrosis markers were notably decreased in the SIL and β-sitosterol groups compared to the CCl4 group (Figure 4A–4E). Furthermore, in mice, β-sitosterol significantly reduced the serum levels of inflammatory markers (LPS, TNF-α, IL-1, and IL-6) compared to CCl_4_ (Figure 4F–4I). Results from LX-2 cells demonstrated that β-sitosterol decreased the protein expression of COL1A1 when compared to the control, indicating its capacity to ameliorate liver fibrosis in both *in vivo* and *in vitro* conditions (Figure 5A).

To examine the effect of β-sitosterol on LX-2 cell activity, a CCK-8 assay was performed, which showed a time-dependent effect (Figure 5B). β-sitosterol was found to induce apoptosis in LX-2 cells, as confirmed by TUNEL staining (Figure 5D). Western blot analysis further confirmed the effects of β-sitosterol treatment, showing a decrease in CASP3 precursor and BCL2 expression, as well as an increase in c-CASP3 and c-CASP8, and a decrease in CASP8 compared to the control (Figure 5C). The findings suggest that β-sitosterol has the potential to cause apoptosis in HSCs.

The collective evidence from IHC, ELISA, cellular assays, and Western blot analysis demonstrate that β-sitosterol can ameliorate liver fibrosis by lowering fibrosis indicators, suppressing inflammation, and modulating proteins involved in the apoptotic pathway, leading to apoptosis in HSCs. By performing a low-dimensional analysis of transcriptome data, WGCNA is employed to find the module most associated with liver injury in mice.

**Figure 6:**
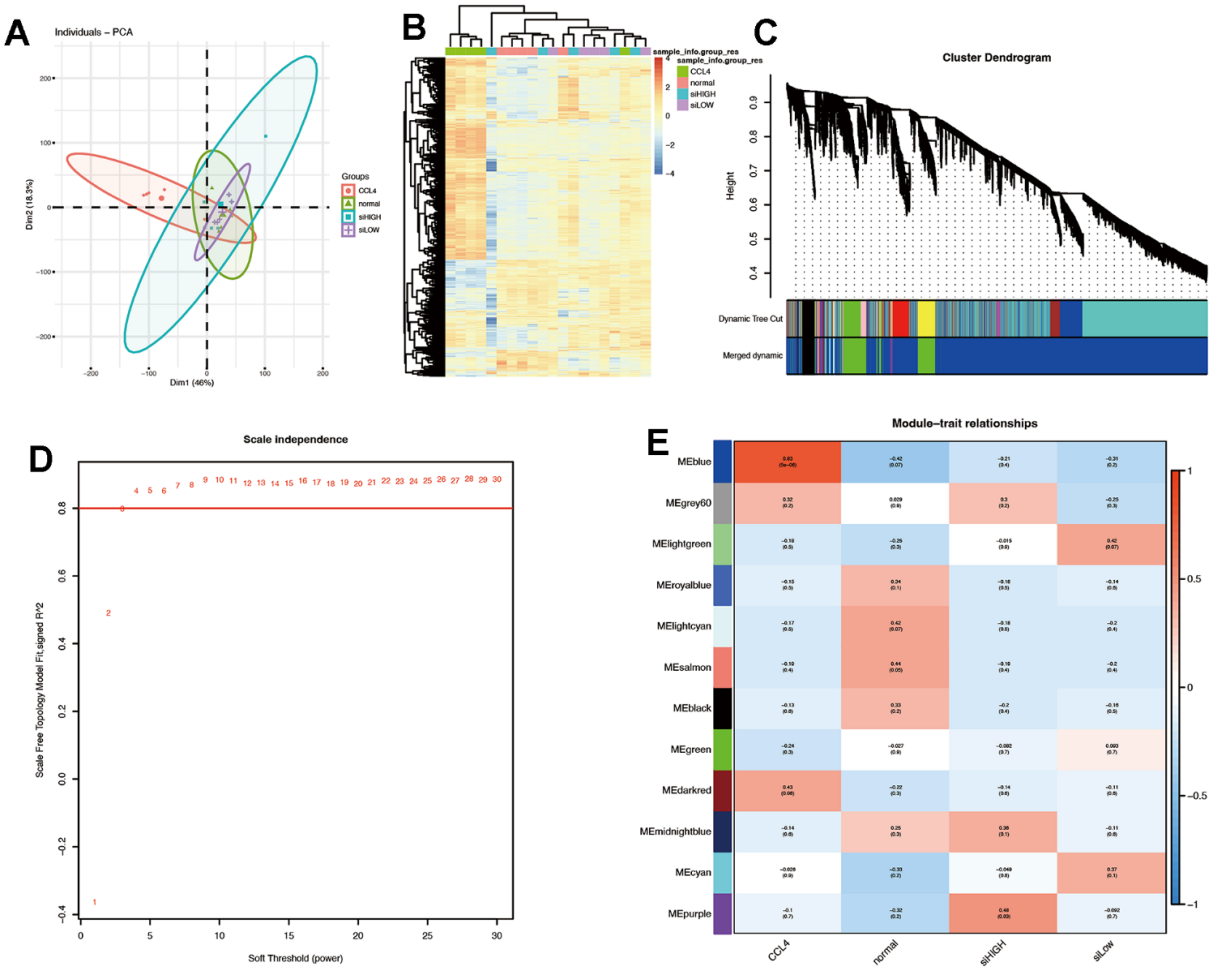
**Quality control of samples was done to determine RNA quantity and weighted gene co-expression network analysis (WGCNA) was performed using an R package of WGCNA for finding modules of highly correlated genes.** (**A**, **B**) PCA plot and heat map. The results suggest that CCL4 is clearly distinguished spatially from the other three groups. (**C**) Gene dendrogram and modules before merging. (**D**) Optimal soft threshold is 3. (**E**) Pearson correlation analysis of the combined modules and individual subgroups. The blue module had the highest correlation coefficient with the CCL4 group.

PCA was employed to reduce the dimension of the pre-processed matrix, and it was observed that the CCl4 model was distinct from the other three datasets (Figure 6A). The heat map analysis further supported these findings (Figure 6B). The WGCNA package was used to identify twelve gene co-expression modules from a dataset consisting of 19,971 genes across 20 samples, as shown in Figure 6C, 6D. The soft threshold was set to three (Figure 6C).

**Figure 7:**
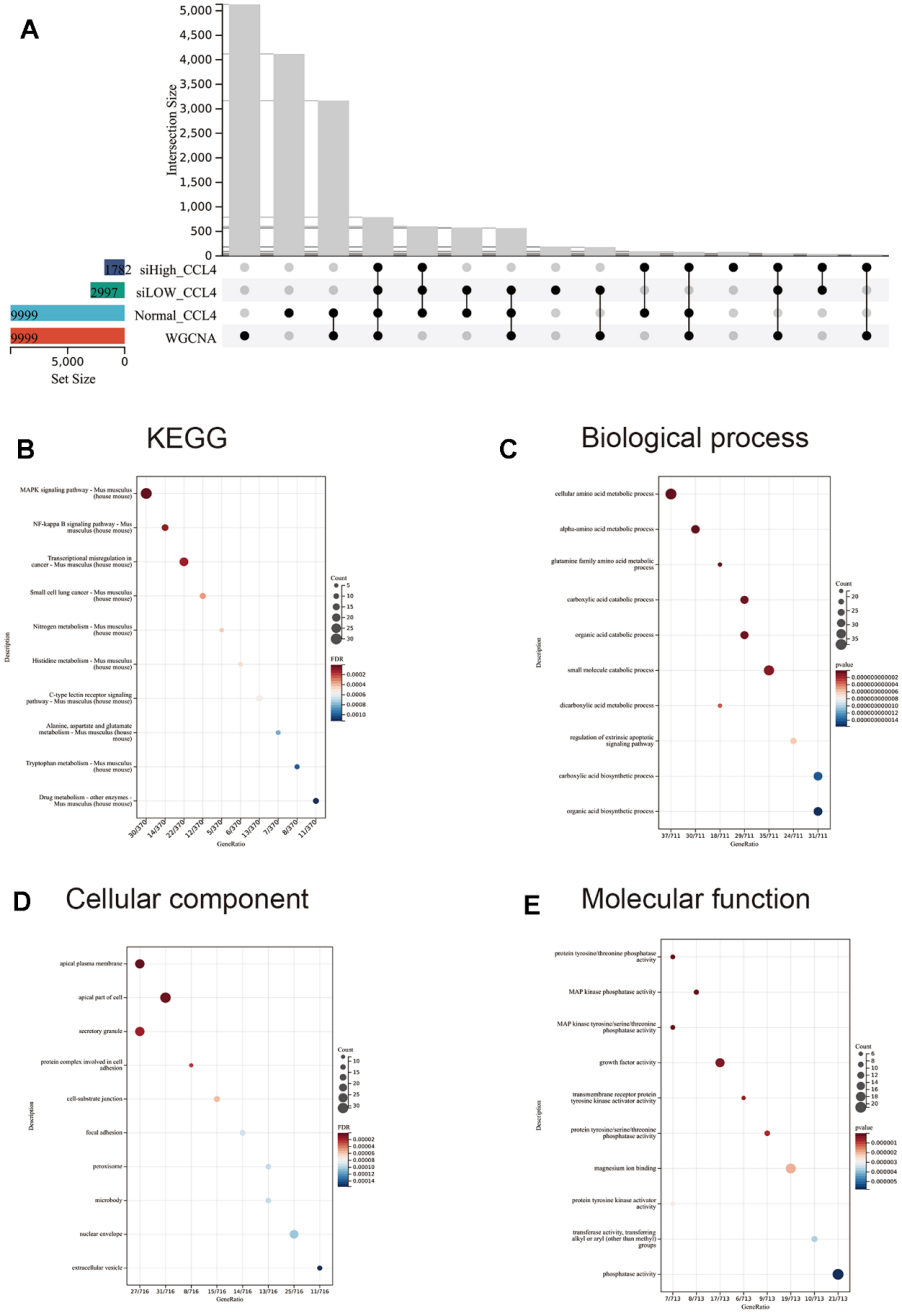
**WGCNA was intersected with the three groups of differential genes taken for GO and KEGG analysis.** (**A**) Upset diagram with a selection of four groups of commonly expressed modules. (**B**–**E**) GO and KEGG analyses were performed according to the modules co-expressed by the four groups. The top 10 significantly enriched (p < 0.05) terms for BP, CC and MF and the top 10 significantly enriched terms for KEGG were selected. The Y-axis represents the number of enrichments for the target and the X-axis represents the GO category of the target gene.

The blue module, which showed the strongest correlation with the CCl_4_ group and negative correlation with the normal, β-sitosterol low and high dose groups was identified as the most significant module (Figure 6E). Based on the differential genes between each group and the control group, this module was crossed with the differentially expressed genes and the resulting genes were analysed using GO and KEGG pathway analysis (Figure 7A).

Investigation uncovered that the differentially expressed genes were linked to a variety of pathways, including MAPK, NF-κB, transcriptional misregulation in cancer, small cell lung cancer, nitrogen metabolism, histidine metabolism, C-type lectin receptor signaling, alanine, aspartate, and glutamate metabolism, tryptophan metabolism, and drug metabolism.

Results of the BP terms analysis indicate that there is a disparity in gene enrichment when it comes to the regulation of amino acid metabolism and extra-apoptotic signalling pathways (Figure 7C). As for the CC terms, the most noteworthy ones were the apical plasma membrane, apical part of the cell, secretory granules, protein complexes involved in cell adhesion, cell-substrate junctions, focal adhesions, peroxisomes, microbodies, nuclear envelopes, and extracellular vesicles (Figure 7D). The main MF terms identified were protein tyrosine/threonine phosphatase activity, MAP kinase phosphatase activity, MAP kinase tyrosine/serine/threonine phosphatase activity, growth factor activity, transmembrane receptor protein tyrosine kinase activator activity, protein tyrosine/serine/threonine phosphatase activity, magnesium ion binding, protein tyrosine kinase activator activity, transferase activity, and phosphatase activity (Figure 7E).

### β-sitosterol reduces NF-κB pathway activation and alleviates liver fibrosis by targeting and regulating MK3

**Figure 8:**
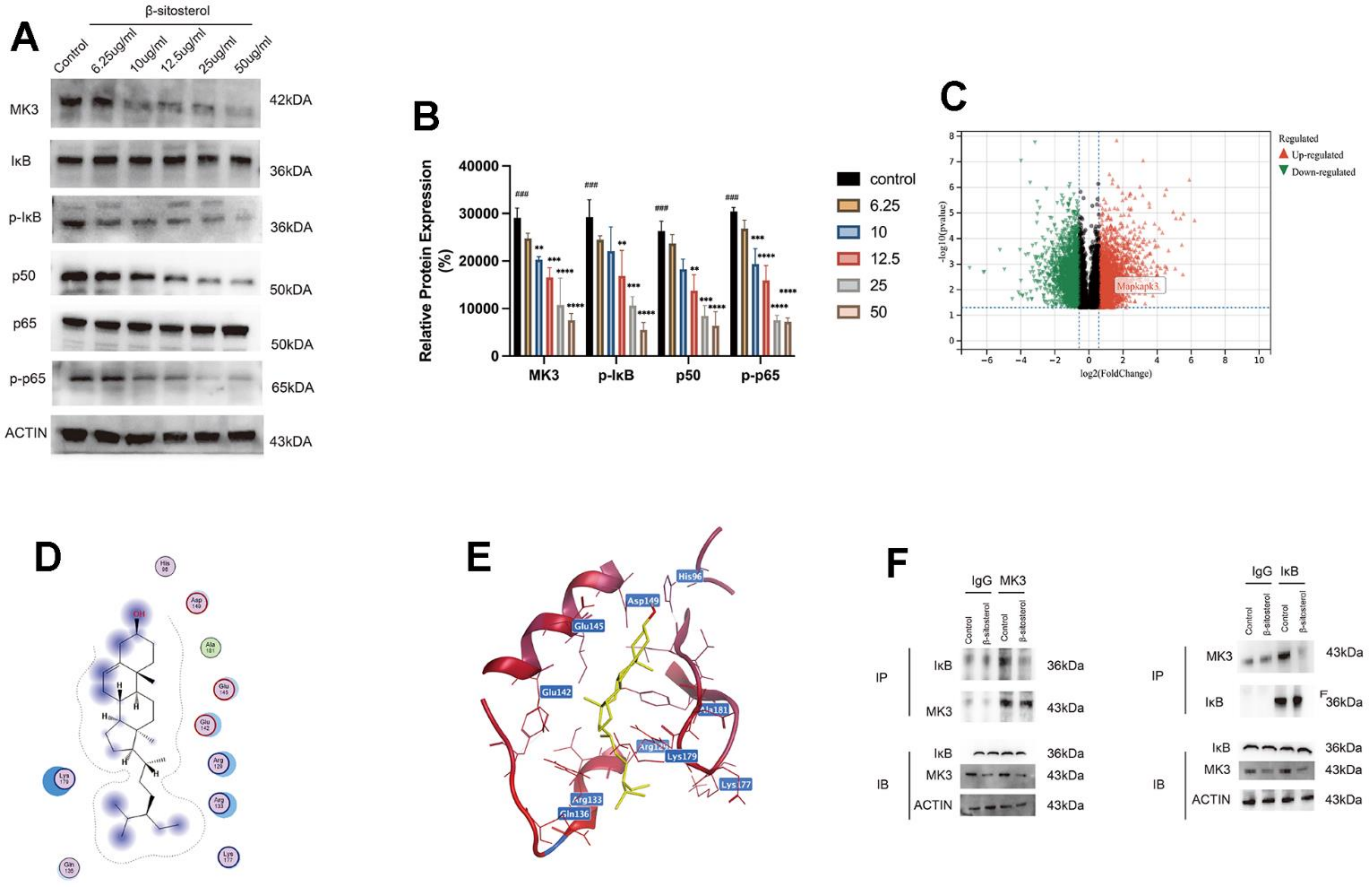
**β-sitosterol inhibits MAPKAPK3 and downstream NF-κB pathway activation in LX-2 cells.** (**A**, **B**) Determination of MAPKAPK3 and NF-κB pathway-related molecules in LX-2 by Western blot. Representative results from three independent replicate experiments are shown (n = 3). Data are expressed as mean ± SD (n = 3). Compared to Control; * P < 0.05, ** P < 0.01, *** P < 0.001, **** P < 0.0001. (**C**) Upregulated and downregulated genes of control and CCL4 groups are plotted by volcano plot. (**D**, **E**) β-sitosterol and MAPKAPK3 molecular docking results. (**F**) CO-IP assay shown that MK3 interacts with IκB, while β-sitosterol reduces MK3 binding to IκB.

KEGG enrichment analysis identified the MAPK and NF-κB signaling pathways as being associated with the genes showing significant differential expression. Additionally, the MF analysis indicated the enrichment of the MAP phosphatase activation pathway among these genes. Given these findings, further investigation focused on these two pathways. Notably, MK3 exhibited a significant elevation in the CCl_4_ group (Supplementary Figure 1), which was subsequently reduced by the administration of low and high doses of β-sitosterol. Therefore, we identified MK3 as a potential target of β-sitosterol (Figure 8C). To validate this observation, we conducted experiments in LX-2 cells. Our findings revealed a progressive decrease in MK3 expression with increasing concentrations of β-sitosterol, indicating a concentration-dependent effect (Figure 8A).

To gain insights into the mechanism by which β-sitosterol can ameliorate liver fibrosis through the modulation of MK3, we referred to our KEGG analysis, which revealed differential gene enrichment in the NF-κB pathway. Our results demonstrated that β-sitosterol reduced the levels of p50, p65, and pp-65 and the phosphorylation of IκB. These findings suggest that β-sitosterol can attenuate NF-κB pathway activation by inhibiting MK3, thereby reducing liver inflammation and fibrosis (Figure 8A, 8B). Furthermore, the molecular docking analysis indicates that the interaction between β-sitosterol and MK3 has a low binding energy, with the lowest binding energy being -6 (Supplementary Table 2). This suggests that MK3 is a target of action of β-sitosterol (Figure 8D, 8E). We then performed CO-IP experiments, which suggested that MK3 can bind to IκB, while β-sitosterol reduces the binding of MK3 to IκB (Figure 8F).

## DISCUSSION

A combination of inflammation and viral damage can cause liver fibrosis, which, if left untreated, can progress to cirrhosis and even liver cancer [26]. Liver fibrosis primarily occurs due to the activation of HSCs triggered by inflammation [2]. Effective management of liver fibrosis involves reducing HSCs and preventing their activation [27]. The apoptosis of aHSCs also prevents inflammatory liver damage, which makes it a crucial aspect of liver fibrosis treatment [28].

RTT is the root of *Ranunculus Ternati* and contains endolipids and sterols as its main constituents [29]. It is commonly used to treat liver conditions, including infection with hepatitis B virus [10]. Network pharmacology and experimental validation were employed in this study to investigate the bioactive components and therapeutic effects of RTT in the treatment of liver fibrosis.

Its main active ingredient β-sitosterol has anti-tumor, anti-oxidant, and anti-inflammatory properties; it is the most important active ingredient of RTT [30–32]. It has been shown to induce apoptosis in ovarian cancer cells, thereby exerting anti-tumor effects [30]. In addition, it alleviated CCl4-induced liver fibrosis in rats, though the underlying mechanism has not yet been elucidated [24].

Network pharmacology analysis identified BCL2, CASP3, and CASP8 as major targets of β-sitosterol in liver fibrosis management. *In vitro* experiments confirmed this finding, and *in vivo* experiments demonstrated that β-sitosterol not only reduced liver fibrosis markers but also decreased liver inflammation. We postulate that β-sitosterol can obstruct the activation of HSCs by diminishing the amounts of inflammatory molecules including IL-1, IL-6, TNF- α, and LPS, and additionally encourage the apoptosis of these cells.

Promoting the apoptosis of HSCs and reducing their activation are essential therapeutic strategies for the management of liver fibrosis. Therefore, β-sitosterol may be a promising treatment option for liver fibrosis.

To investigate β-sitosterol-induced apoptosis in HSCs, we performed RNA-seq on mice liver samples. WGCNA analysis revealed a considerable rise in the number of genes that were differentially expressed in both the MAPK and NF-κB pathways, both of which are heavily linked to liver fibrosis. The MAPK pathway has a role in relaying TGF-β signals, thus aiding the progression of liver fibrosis [33].

**Figure 9:**
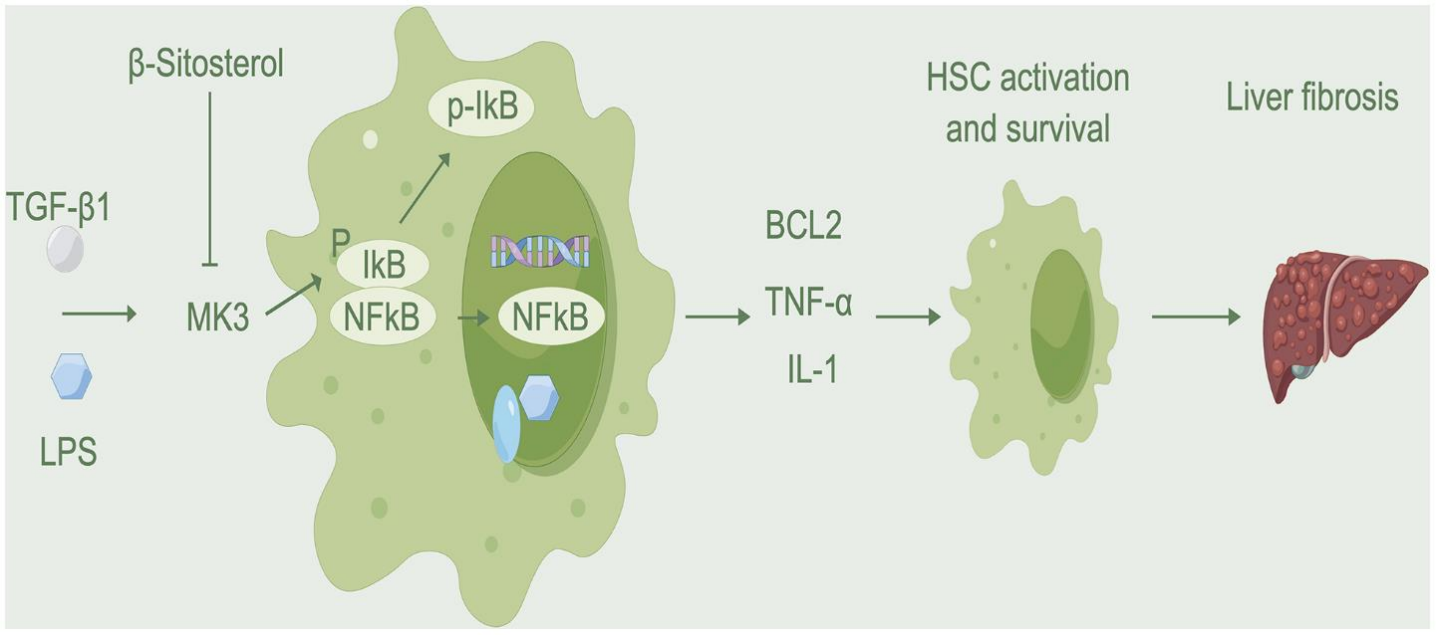
**Mechanism diagram.** β-sitosterol inhibits the binding of MK3 to IκB, reduces the activation of MK3 and IκB, promotes HSC apoptosis, and alleviates liver fibrosis.

The NF-κB pathway involved in the progression of liver fibrosis by activating and transcribing genes such as BCL2 and IL-1, which allow aHSCs to survive and maintain qHSC activation [34–36]. Both pathways are essential for liver fibrosis and have a direct correlation to its progression. Results from Western blot analysis indicated that β-sitosterol was capable of hindering the phosphorylation and degradation of IκBα, a typical representative of the IκB family, as well as the phosphorylation of p65. Usually, the p65/p50 heterodimer is associated with IκBα and remains inactive. The phosphorylation of IκBα protein, which is caused by rapid signaling, is essential for NF-κB p65 to move into the nucleus, bind to DNA and then IκBα is broken down [37]. MK3, a component of the MAPK pathway, exhibits significant changes. Hexokinase 2 can directly phosphorylate and activate IκB proteins [38], leading to their degradation through auto-ubiquitination and regulating the NF-κB pathway. We hypothesized that MK3 may be responsible for phosphorylating and degrading IκB. Experimental findings confirmed that β-sitosterol targets MK3, inhibiting this process. This process suppresses the NF-κB pathway, leading to a decrease in BCL2 transcription and the promotion of apoptosis in HSCs. Molecular docking analysis of the interaction between β-sitosterol and MK3 revealed a low binding score energy (-6.0), indicating a strong binding between the two. Results from CO-IP assays indicate that MK3 binds directly to IκB, resulting in the degradation of IκB phosphorylation. On the other hand, β-sitosterol reduces the binding of MK3 to IκB and thus reduces the activation of the NK-κB pathway. It has been suggested that MK3 is a major factor in the development of liver fibrosis, and β-sitosterol could be used to target MK3, thereby reducing the phosphorylation of IκB and the activation of the NK-κB pathway (Figure 9). β-sitosterol has the potential to be therapeutically effective in reducing liver fibrosis, with MK3 being a key target for this purpose.

This experiment has some limitations. For example, we did not investigate the region and site at which MK3 phosphorylates IκB. Further studies are needed to analyze the interaction between MK3 and IκB, with the aim of discovering new therapies for the management of liver fibrosis.

In summary, RTT can alleviate liver damage and reduce inflammatory mediators. Our hypothesis is that β-sitosterol inhibits the NF-κB pathway through MK3 modulation, reducing the binding to IκB, and encourages the apoptosis of HSCs.

## CONCLUSIONS

In conclusion, the active ingredient of RTT, β-sitosterol, alleviates liver fibrosis by reduction of inflammatory mediator release, and targeting MK3, while reducing the binding of MK3 to IκB and reducing the activation of the NK-κB pathway, thereby promoting apoptosis of HSC. RTT is a promising drug for liver fibrosis.

## Supplementary Material

Supplementary Figure 1

Supplementary Tables
