# Supplementary material for: Network pharmacology and transcriptomic profiling elucidate the therapeutic effects of Ranunculus ternatus Thunb on liver fibrosis via MK3-NF-κB inhibition
Source: Aging (Albany NY). 2024 Mar 8;16(5):4759–77. doi: 10.18632/aging.205629 (PMC10968670; doi:10.18632/aging.205629)
Supplement: Supplementary Figure 1 [file aging-16-205629-s001.pdf]

SUPPLEMENTARY FIGURE

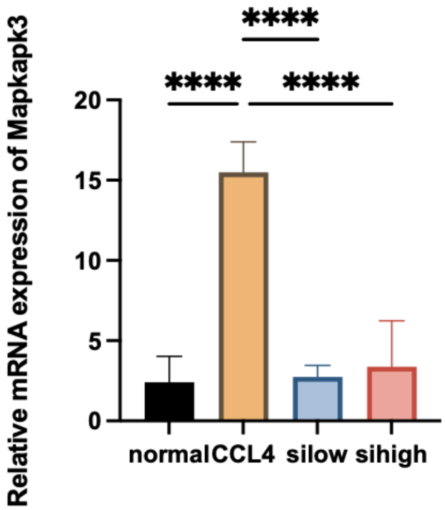

Supplementary Figure 1. MAPKAPK3 was differentially expressed in the normal, CCL4 group, β-sitosterol low dose group and β-sitosterol high dose group. Representative results of at least six independent experiments (biological replicates) are shown in all panels. * P <0.05, ** P <0.01, *** P <0.001, **** P <0.0001.
