# Supplementary material for: Network pharmacology and transcriptomic profiling elucidate the therapeutic effects of Ranunculus ternatus Thunb on liver fibrosis via MK3-NF-κB inhibition
Source: Aging (Albany NY). 2024 Mar 8;16(5):4759–77. doi: 10.18632/aging.205629 (PMC10968670; doi:10.18632/aging.205629)
Supplement: Supplementary Tables [file aging-16-205629-s002.pdf]

SUPPLEMENTARY TABLES

Supplementary Table 1. *Ranunculus ternatus* Thunb main active ingredients and related indicators.

Mol ID	Molecule name	OB	DL
MOL011319	Truflex OBP	43.74	0.24
MOL011328	Stigmasta-4,6,8	48.02	0.77
MOL011330	vittadinoside_qt	43.83	0.76
MOL001494	Mandenol	42.00	0.19
MOL001973	Sitosteryl acetate	40.39	0.85
MOL000242	7-O-Methyleriodictyol	56.56	0.27
MOL000358	beta-sitosterol	36.91	0.75
MOL000449	Stigmasterol	43.83	0.76
MOL005438	campesterol	37.58	0.71
MOL006772	poriferasterol monoglucoside_qt	43.83	0.76
MOL000953	CLR	37.87	0.68

Supplementary Table 2. Five sites of β-sitosterol binding to MK3 and binding energy

Mol	S
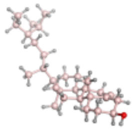	-6.0349
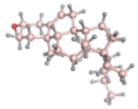	-5.9025
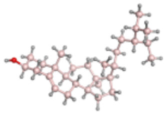	-5.6048
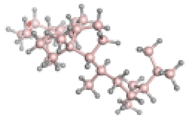	-5.5770
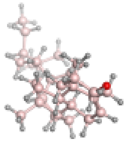	-5.5684
